# Phage Display Screening for Tumor Necrosis Factor-****α****-Binding Peptides: Detection of Inflammation in a Mouse Model of Hepatitis

**DOI:** 10.1155/2013/348409

**Published:** 2013-02-26

**Authors:** Coralie Sclavons, Carmen Burtea, Sébastien Boutry, Sophie Laurent, Luce Vander Elst, Robert N. Muller

**Affiliations:** ^1^Department of General, Organic and Biomedical Chemistry, NMR and Molecular Imaging Laboratory, University of Mons, Mendeleïev Building, 19 Avenue Maistriau, 7000 Mons, Belgium; ^2^Center for Microscopy and Molecular Imaging (CMMI), 8 Rue Adrienne Bolland, 6041 Gosselies, Belgium

## Abstract

TNF-**α** is one of the most abundant cytokines produced in many inflammatory and autoimmune conditions such as multiple sclerosis, chronic hepatitis C, or neurodegenerative diseases. These pathologies remain difficult to diagnose and consequently difficult to treat. The aim of this work is to offer a new diagnostic tool by seeking new molecular probes for medical imaging. The target-specific part of the probe consists here of heptameric peptides selected by the phage display technology for their affinity for TNF-**α**. Several affinity tests allowed isolating 2 peptides that showed the best binding capacity to TNF-**α**. Finally, the best peptide was synthesized in both linear and cyclic forms and tested on the histological sections of concanavalin-A-(ConA-)treated mice liver. In this well-known hepatitis mouse model, the best results were obtained with the cyclic form of peptide 2, which allowed for the staining of inflamed areas in the liver. The cyclic form of peptide 2 (2C) was, thus, covalently linked to iron oxide nanoparticles (magnetic resonance imaging (MRI) contrast agent) and tested in the ConA-induced hepatitis mouse model. The vectorized nanoparticles allowed for the detection of inflammation as well as of the free peptide. These *ex vivo* results suggest that phage display-selected peptides can direct imaging contrast agents to inflammatory areas.

## 1. Introduction

Tumor necrosis factor alpha (TNF-*α*) is a proinflammatory cytokine produced in many inflammatory and autoimmune diseases such as rheumatoid arthritis, Crohn's disease, multiple sclerosis, or chronic hepatitis C [1, 2]. TNF-*α* is produced by different cell types including macrophages, monocytes, T-cells, smooth muscle cells, adipocytes, and fibroblasts. This cytokine is also implicated in the diseases of the central nervous system like Alzheimer's and Parkinson's diseases [3], where it can be produced by several cell populations, including microglia, astrocytes, endothelial cells, Th1 lymphocytes and neurons.

Mature TNF-*α* is secreted as a 157-amino acid form [4] with a molecular weight of 17 kDa [5]. Before being released from cells, TNF-*α* is anchored in the plasma membrane as a 26 kDa precursor containing both hydrophobic and hydrophilic regions [6]. The 17 kDa form of TNF-*α* is excised from the integral transmembrane precursor by proteolytic cleavage mediated by the tumor necrosis factor alpha converting enzyme (TACE) [7]. Soluble and transmembrane TNF-*α* are produced by cells as homotrimers that bind to two kinds of receptors, TNF-RI and TNF-RII (tumor necrosis factor receptor type I, p55; type II, p75, resp.), which are present in the membrane of all cell types except erythrocytes.

TNF-*α*, which is one of the most abundant cytokines involved in apoptotic and inflammatory pathways, has both desirable and undesirable effects. Tumor necrosis induction and mediation of the immune response to bacterial, viral, and parasitic invasions are beneficial functions of TNF-*α* [6]. It is also an acute phase protein that initiates a cascade of cytokines and increases vascular permeability, thereby recruiting macrophages and neutrophils to a site of infection. However, TNF-*α* can also have pathological consequences such as promoting the growth of some tumor cell types. It also plays an important role in the chronic inflammation that occurs in various pathologies and has been identified as the major mediator in various autoimmune diseases [8, 9]. TNF-*α* thus represents a good marker of inflammatory events.

Phage display is a high-throughput screening (HTS) method. It is an effective way of selecting target-specific proteins and peptides that can be synthesized and linked to an imaging reporter for diagnostic use. This technique can be used to identify peptides or antibodies capable of interacting with inflammatory mediators [10, 11].

In the present work, a heptapeptide phage display library was screened against TNF-*α*, as a first step in the development of new molecular imaging tools for detecting inflammation in chronic inflammatory pathologies and diseases of uncertain diagnosis. Peptide specificity for the cytokine was tested on histological liver sections of mice treated with concanavalin A (ConA), which is a mitogenic lectin known to stimulate lymphocytes to produce lymphokines [12] mediating chronic inflammation processes [13] and inducing severe injury to hepatocytes [14]. ConA induces an autoimmune hepatitis due to massive CD4+ lymphocyte activation and infiltration into the liver parenchyma leading to the secretion of the proinflammatory cytokines TNF-*α*, interferon-*γ* (IFN*γ*), interleukin (IL)-2, IL-6, granulocyte macrophage-colony stimulating factor, and IL-1 [15]. The phage display-selected heptapeptide was then grafted to iron oxide nanoparticles (known for their use as MRI contrast agents). This new peptide was thus set up as an iron oxide-based probe and used to detect inflammatory process on histological liver sections prior to further *in vivo* MRI tests.

## 2. Methods

### 2.1. Phage Display

#### 2.1.1. The Biopanning of PhD-C7C Phage Display Library against TNF-*α*

The well of an ELISA microtiter plate was coated with 100 *μ*L of target solution (0.1 mg/mL of human TNF-*α* (GenScript Corporation, Piscataway, USA) in 0.1 M NaHCO_3_ buffer, pH 8.6) by overnight incubation at 4°C in a humid chamber. The next day, the target solution was removed and replaced by the blocking buffer (Bovine Serum Albumin, 5 mg/mL; 0.1 M NaHCO_3_, pH 8.6, NaN_3_ 0.02%) for 2 hours and finally washed with Tris-buffered saline (TBS) supplemented with 0.1% Tween-20 (TBS-T, 50 mM Tris-HCl, 150 mM NaCl, pH 7.4). After negative selection on a BSA-coated well, the phage library (2 × 10^11^ phages in 100 *μ*L of TBS-T 0.1%; PhD-C7C, New England Biolabs Inc., Westburg B. X., Leusden, The Netherlands) was incubated during 1 hour at 37°C with the target. After rinsing, TNF-*α*-bound phages were eluted with 0.2 M glycine-HCl buffer (pH 2.2) complemented with 0.1% BSA and then neutralized with 1 M Tris-HCl buffer (pH 9.1).

Eluted phages were amplified during 4 h30 via *Escherichia coli* (ER2738 host strain, New England Biolabs Inc.) infection. Amplified phages were collected by two precipitations at 4°C in PEG-NaCl solution (20% polyethylene glycol-8000, 2.5 M NaCl). The phage pellet was finally solubilized in a TBS buffer solution (50 mM Tris-HCl, 150 mM NaCl, pH 7.5). This succession of steps was repeated 4 times and constitutes a biopanning round. The selective pressure was increased during the third and the fourth rounds of biopanning by increasing the Tween-20 concentration in the incubation and rinsing buffers to 0.3% and 0.5%, respectively, and by reducing the incubation time to 45 min and 30 min, respectively.

*E. coli* was grown on a selective medium containing isopropyl-beta-D-thiogalactoside (IPTG) (ICN Biomedical Inc., Brussels, Belgium) and 5-bromo-4-chloro-3-indolyl-beta-D-galactopyranoside (Xgal) (Sigma-Aldrich, Bornem, Belgium). The phage genome contains a part of the LacZ gene that confers to bacteria the ability to produce *β*-galactosidase, which reacts with the X-gal substrate and results in a blue plaque staining. Phage titer is, thus, achieved by counting phage-infected *E. coli* (blue-colored) colonies after each biopanning round.

#### 2.1.2. Sequencing of Selected Phage Clones

The genome sequencing of selected phage clones was based on the Sanger method which uses dideoxynucleotides triphosphate as DNA chain terminators. Briefly, DNA is extracted by the phenol/chloroform extraction procedure [16] and denatured by several heating cycles. Virus genome is sequenced by using a Start Mix solution (Beckman Coulter, Analis, Namur, Belgium) and a 20-base primer (5′-CCCTCATAGTTAGCGTAACG-3′, New England Biolabs Inc.) located 96 nucleotides upstream to the inserted peptide-encoding sequence. The Start Mix solution is the sequencing reaction buffer containing 4 ddNTPs, 4 dNTPs, and the DNA polymerase enzyme.

The DNA sequence was analyzed on a CEQ 2000 XL DNA Analysis System (Beckman Coulter, Analis). The sequence reading was performed automatically using the JaMBW 1.1 software (http://bioinformatics.org/JaMBW/).

#### 2.1.3. Evaluation of the Affinity of Selected Clones for the Target

*(a) Estimation of the Apparent Dissociation Constant *(*K*_d_*)* between the Phage Clones and TNF-*α*.* The *K*_d_* of phage clones against their target was assessed by the ELISA (enzyme-linked immunosorbent assay) method, using a range of phage dilutions incubated with the specific target.

ELISA plates were treated overnight with the target solution (human TNF-*α*, 0.01 mg/mL, at 4°C). The next day, the target solution was replaced by the blocking solution as described above. Plates were then incubated for 2 h at 37°C with serial phage dilutions (6.5 × 10^−11^ – 3.3 × 10^−8^ M) prepared in PBS containing 0.3% Tween 20. After rinsing the plate 6 times with the same buffer, phages were detected with a peroxidase-conjugated anti-M13 monoclonal antibody (Amersham Pharmacia Biotech Benelux, Roosendaal, The Netherlands) diluted 1 : 5000 in the blocking buffer. The peroxidase detection was performed using 2,2′-azino-bis[3-ethylbenztiazoline-6-sulfonic acid, diammonium salt] (ABTS, Sigma-Aldrich; 22 mg in 100 mL of 50 mM sodium citrate, pH 4.0) supplemented with 0.05% H_2_O_2_. After 30–60 min of incubation at room temperature, the OD_405_ values were measured on a microplate reader (Stat-Fax-2100, Awareness Technology Inc., Fisher Bioblock Scientific, Tournai, Belgium) for  *K*_d_* evaluation.

*(b) Competition Experiments with an Anti-TNF-*α* Antibody.* Competition experiments were performed in the same manner as the *K*_d_* experiments except that clones were set in competition with an anti-TNF-*α* antibody (R&D Systems Inc., Abington, UK) in dilutions ranging from 1.3 × 10^−9^ to 6.66 × 10^−7^ M in a PBS solution. Plates were first incubated for 30 min with 50 *μ*L/well of antibody dilutions before a one-hour incubation with 50 *μ*L of phage solutions at the  *K*_d_*  concentration. The IC_50_ of the anti-TNF-*α* antibody is the antibody concentration required to inhibit by 50% the interaction between phages and TNF-*α*.

#### 2.1.4. Synthesis of Biotinylated Peptides

Peptides displayed on the surface of selected phage clones were synthesized in a biotinylated form by NeoMPS (Polypeptide group, Strasbourg, France). Biotinylated peptides were synthesized in two forms: a linear form (L) and a cyclic (C) form.

#### 2.1.5. The Evaluation of the Affinity of Selected Peptides for the Target

The affinity of the selected peptides was evaluated by ELISA as for the phage clones (see Section 2.1.3). The target was immobilized overnight on a 96-well plate at 4°C before a 2-hour incubation with a protein-free blocking buffer solution (PFBB, Pierce, Aalst, Belgium) and then with a range of peptide dilutions: 10^*‒*3^ M to 10^*‒*6^ M. The target-bound biotinylated peptides were detected using ABC (Avidin Biotin Complex) and ABTS solutions as described above. The dissociation constant was estimated from the inflection point of the curve.

### 2.2. The Physicochemical Characterization of the 2C Peptide-Grafted Nanoparticles

The phage display-selected 2C peptide was grafted to ultrasmall nanoparticles of iron oxide (USPIO). The particles were pegylated with the amino-PEG 750 (Fluka, Bornem, Belgium) to avoid the rapid uptake of USPIO by macrophages and to prolong the blood circulation time.

Peptide-USPIO-PEG was prepared in our laboratory from previously described nanoparticles bearing carboxylated groups on their surface [17–19]. USPIOs were functionalized in two successive steps: first, they were functionalized with the peptide, and, second, they were coupled to an amino-PEG 750. Briefly, 19 mg of the peptide and 6 mg of EDCI (1-ethyl-3-(3-dimethylaminopropyl) carbodiimide) were added to 15 mL of nanoparticles ([Fe] = 0.175 Mol. Peptide/Fe molar percent is 0.02%), and the mixture was stirred overnight. The nanoparticle suspension was ultrafiltrated on a 30 kD membrane. Amino-PEG 750 (0.503 g) and EDCI (0.327 g) were added to the nanoparticles (PEG/Fe molar percent is 2%) and the reaction was stirred during 17 h. The mixture was ultrafiltrated on a 30 kD membrane to remove low-molecular material.

NMRD (Nuclear Magnetic Resonance Dispersion) profiles were recorded at 37°C on a Fast Field Cycling Relaxometer (Stelar, Mede, Italy) over a magnetic field range from 0.24 mT to 0.24 T. Additional longitudinal (R_1_) and transverse (R_2_) relaxation rate measurements at 0.47 T and 1.41 T were obtained on Minispec MQ 20 and MQ 60 spin analyzers (Bruker, Karlsruhe, Germany), respectively. The fitting of the NMRD profiles by a theoretical relaxation model [20] allowed the determination of the crystal radius (*r*) and the specific magnetization (*M*_*S*_).

Hydrodynamic size measurement was carried out by photon correlation spectroscopy (PCS) on a Zêtasizer Nanoseries ZEN 3600 (Malvern, UK).

Total iron concentration was determined by proton (^1^H) relaxometry at 20 MHz and 310 K after microwave digestion (MLS-1200 MEGA, MILESTONE, Analis, Namur, Belgium).

### 2.3. Animals and Treatments

Adult male mice C57BL/6JOlaHsd (6 months old, Harlan Laboratories B.V., The Netherlands) were used.

All animals were housed in plastic cages under a 12 h light/12 h dark cycle and had free access to food and water. Ambient temperature was maintained at 25 ± 2°C. Animals were maintained and treated in compliance with the guidelines specified by the Belgian Ministry of Trade and Agriculture.

Adult mice (*n* = 6) received a single i.p. injection of 20 mg/Kg of ConA [21], a lymphocyte T activator producing inflammation in mice liver.

### 2.4. Immunohistochemistry

#### 2.4.1. Tissue Preparation

Mice were sacrificed 2 hours after intravenous injection of ConA, and their livers were fixed for 24 h in 4% paraformaldehyde. After fixation, the liver was dehydrated by several alcohol and butanol soakings and the organ was subsequently embedded in paraffin according to standard procedures. Sections of 4-micrometer thickness were cut for histological tests.

#### 2.4.2. TNF-*α* Detection by a Polyclonal Antibody in Liver Histological Sections

Liver sections were dewaxed and rehydrated by several washes in toluene and alcohol. They were subsequently treated for 5 minutes with 2% H_2_O_2_ in order to block endogenous peroxidases. Endogenous biotins and nonspecific epitopes were blocked successively with an avidin/biotin and casein solution before proceeding to incubate the liver sections overnight with a rabbit anti-mouse TNF-*α* polyclonal antibody (1 : 100, Abcam, Paris, France). The next day, liver sections were incubated for 1 hour with a biotinylated goat anti-rabbit antibody (Vector Labs, Brussels, Belgium). Reactive sites were detected with the avidin-biotin peroxidase complex (ABC, Vector Labs) and revealed with DAB (3,3′-diaminobenzidine, Sigma-Aldrich, Bornem, Belgium) and 0.02% of H_2_O_2_ prepared in PBS. Sections were washed in distilled water to stop the reaction. Counterstaining of liver tissue was performed with Luxol fast blue before mounting in a permanent medium.

#### 2.4.3. TNF-*α* Detection by 2C and 2L Peptides in Liver Histological Sections

TNF-*α* detection by the phage display-selected 2C and 2L peptides was carried out with the same protocol as for anti-TNF-*α* polyclonal antibody (see Section 2.4.2). Briefly, liver sections were incubated overnight with the biotinylated peptides (20 *μ*M). Reactive sites were detected with the ABC complex and DAB staining.

#### 2.4.4. TNF-*α* Detection by 2C Peptide-USPIO-PEG in Liver Histological Sections

The selected peptide (2C) was grafted to USPIO-PEG in order to produce a vectorized MRI contrast agent for the specific targeting of injured areas. The specificity of the 2C peptide-USPIO-PEG was tested on liver sections. Slices were deparaffinated in toluene and rehydrated in alcohol. Sections were then incubated overnight with 30 mM of 2C peptide-USPIO-PEG at 4°C before being immersed for 30 minutes in Prussian blue reagent (5% potassium ferrocyanide in PBS-HCl 2% solution) for the iron staining. Slides were counterstained with eosin during 8 minutes before mounting in a permanent medium.

## 3. Results

### 3.1. Characterization of the Selected Phage Clones

#### 3.1.1. TNF-*α* Affinity

The screening of a cyclic heptamer peptide library displayed by the phage coat allowed for the selection of 48 phage clones from round 3 and 50 phage clones from round 4 of the selection-amplification procedure. Therefore, 98 phages presenting an affinity for the target were collected.

Nineteen clones from round 3 and 23 clones from round 4 were chosen for their higher affinity for TNF-*α* and their lower affinity for BSA coated plates (TNF-*α*/BSA = 2.9 ± 0.7 [mean value ± SEM ] for round 3 clones and TNF-*α* /BSA  = 3.1 ± 0.5 [mean value ± SEM ] for round 4 clones).

**Figure 1:**
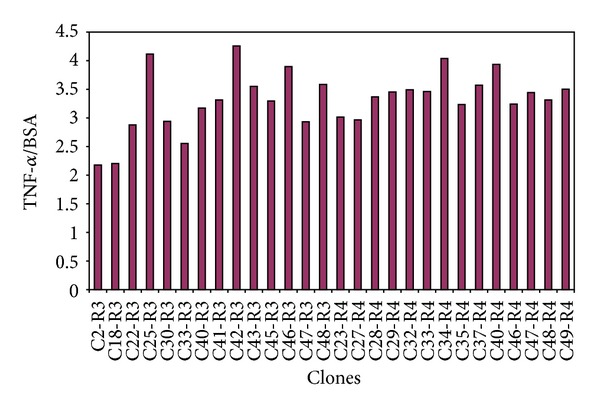
Affinity of the 28 selected clones for the TNF-*α* cytokine. Results are presented as a ratio between the clones' affinity for TNF-*α* and that for BSA. Clones show 4-times higher affinity for TNF-*α* than for BSA. The phage clones were selected after the biopanning rounds 3 and 4.

Based on these results, 28 clones with higher affinity were, thus, selected for further characterization (Figure 1).

#### 3.1.2. Peptide Sequence and Biochemical Parameters of the Selected TNF-*α* Binding Clones

**Figure 2:**
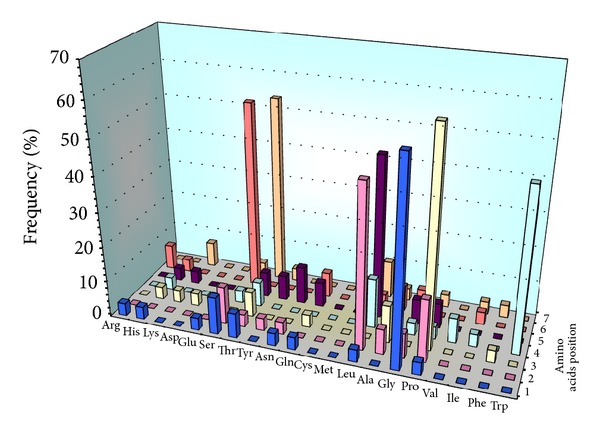
The analysis of the amino acid frequency in each position of the heptapeptide sequences. The 5 first positions are mainly occupied by hydrophobic amino acids while the last two positions are mainly occupied by hydrophilic amino acids.

The DNA of the 28 phage clones selected for their affinity to the TNF-*α* cytokine was sequenced. Fifteen different peptide inserts were identified. Peptide sequences mainly contained hydrophobic amino acids (61%), that is, glycine, leucine, proline, and tryptophan, occupying the first 5 positions in the peptide (Figure 2). Hydrophilic amino acids with a hydroxyl function (i.e., serine and threonine) are less well represented (39%) than hydrophobic amino acids and are most predominant in the last two positions.

Among the fifteen different peptides that were identified from the 28 determined peptide sequences, the cyclic peptides C-GLPWLST-C and C-GPAVYMK-C were found 13 and 2 times, respectively (Table 1).

Two biochemical parameters of these peptides were theoretically estimated by using the EXPASY ProtParam tools (http://www.expasy.ch/tools/protparam.html).

The predicted half-life is the time required for half of the intravenously injected peptide dose to be degraded. The peptides' half-life must be long enough to allow specific interactions between peptides and their targets to take place (e.g., during MRI scans with the peptides linked to contrast agents). The half-life of peptides ranges between 0.8 and 30 hours. The longest predicted half-lives (20–30 hours) were found for the following peptides: C-PATLTSL-C, C-GPAVYMK-C, C-GLPWLST-C, and C-GSKTQAP-C (Table 1).

The isoelectric point (pI) provides information that is important for further applications in physiological conditions. Indeed, an electrical charge borne by the peptide at physiological pH can change its biodistribution *in vivo*. A change of the peptide charge can also affect the affinity for the target and consequently that of the conjugated imaging agent. The pI values of the 28 selected peptides were found to vary from 3.8 to 8.08. Eight of them would be in an ionized state at a pH near the physiological pH of 7.4 (Table 1, bold text).

#### 3.1.3. Estimation of the *K*_d_* between the Phage Clones and TNF-*α*

The *K*_d_* value was estimated for 15 phage clones. One clone was chosen to represent the sequences that were found several times. The clone that showed the best *K*_d_* value is C30-R3 (clone 30 from round 3, C-PATLTSL-C, *K*_d_* = 2.05 × 10^−11^ M). The lowest affinity was found for clone C47-R4 (clone 47 from round 4, C-NNPLKSL-C, *K*_d_* = 9.89 × 10^−6^ M). All the other clones were found to have a *K*_d_* value lower than 10^−8^ M.

**Figure 3:**
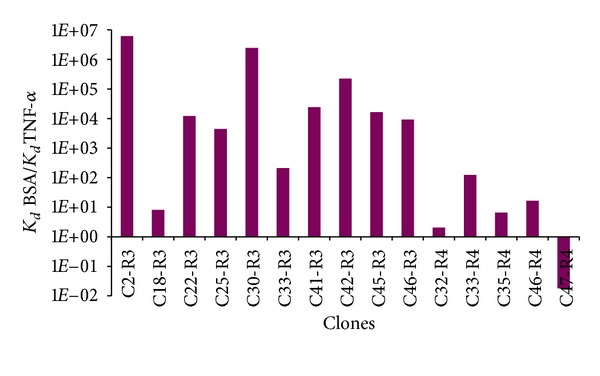
Affinity of the 15 selected phage clones. Clones having the best affinity are the C2-R3 and C30-R3 clones with a *K*_*d*_  BSA/*K*_*d*_  TNF-*α* of 6 × 10^6^ and 4.2 × 10^6^, respectively.

The best clones were chosen on the basis of the ratio *K*_d_* BSA/*K*_d_* TNF-*α* to be tested in competition with an anti-TNF-*α* antibody (Figure 3). Seven phage clones were thus selected for these competition tests: C2-R3 (C-GSKTQAP-C), C22-R3 (C-STPHNLG-C), C30-R3 (C-PATLTSL-C), C41-R3 (C-RPPIGAF-C), C42-R3 (C-TSQSQHM-C), C45-R3 (C-GPAVYMK-C), and C46-R3 (C-QGDLPGY-C). The highest affinity clones were C2-R3 and C30-R3, with a *K*_*d*_ BSA/*K*_*d*_ TNF-*α* ratio of 6 × 10^6^ and 4.2 × 10^6^, respectively; the clone showing the lowest affinity was again C47-R4, with a *K*_*d*_ BSA/*K*_*d*_ TNF-*α* ratio of 0.02.

#### 3.1.4. Competition Experiments with an Anti-TNF-*α* Antibody

The objective of *this* test is to set up a competition between every phage clone and another ligand of the target to calculate an inhibition constant value (IC_50_). A high IC_50_ value indicates that a significant concentration of antibody is necessary to displace phages from their binding sites on the target.

The best phage clone-target interaction was found for C2-R3 and C30-R3 (IC_50_ values of 1.12 × 10^−7^ M and 1.27 × 10^−7^ M, resp.).

**Figure 4:**
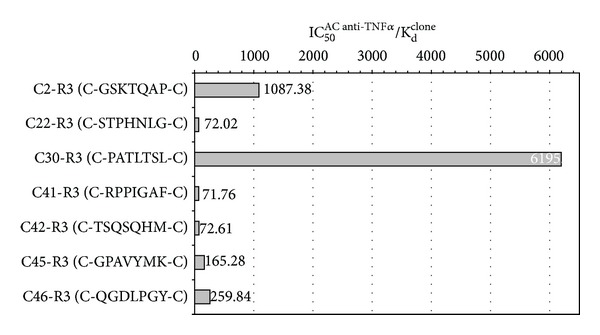
IC_50_^AC⁡  anti-TNF-*α*^/*K*_*d*_^clone^ ratio of the 7 phage clones selected for their high *K*_*d*_ BSA/*K*_*d*_ TNF-*α* ratio. Clones C2-R3 and C30-R3 have the best affinity for the target (IC_50_^AC⁡  anti-TNF-*α*^/*K*_*d*_^clone^ ratio values of 1087 and 6195, resp.). These results are in agreement with the previous ones.

Subsequently, the best phage clones were selected on the basis of their IC_50_^Ab  anti-TNF-*α*^/*K*_*d*_^clone^ratio, which reflects the highest target affinity of a phage clone (Figure 4). The clones C2-R3 and C30-R3 showed the highest ratio (1087 and 6195, resp.).

### 3.2. The Characterization of the Phage Display-Selected Peptides

#### 3.2.1. The Estimation of *K*_d_* of the Selected Peptides for TNF-*α*

The *K*_d_* values of cyclic and linear biotinylated peptides (C-)GSKTQAP(-C) (peptide 1) and (C-)PATLTSL(-C) (peptide 2) were evaluated. (C-)PATLTSL(-C) showed a better affinity for the target than (C-)GSKTQAP(-C). Linear and cyclic forms of GSKTQAP weakly interact with TNF-*α* and the curve does not reach saturation. In comparison with the GSKTQAP peptide, the linear and cyclic forms of the PATLTSL peptide (resp., 2L and 2C peptides) have a good ability to interact with the target (*K*_d_* values of 1.79 × 10^−4^ and 1.12 × 10^−4^, resp.). Both linear and cyclic forms show only a weak interaction with the PFBB coated plate; the *K*_d_* values could not be estimated.

### 3.3. The Physicochemical Characterization of the 2C Peptide Grafted to the USPIO-PEG Nanoparticles

**Figure 5:**
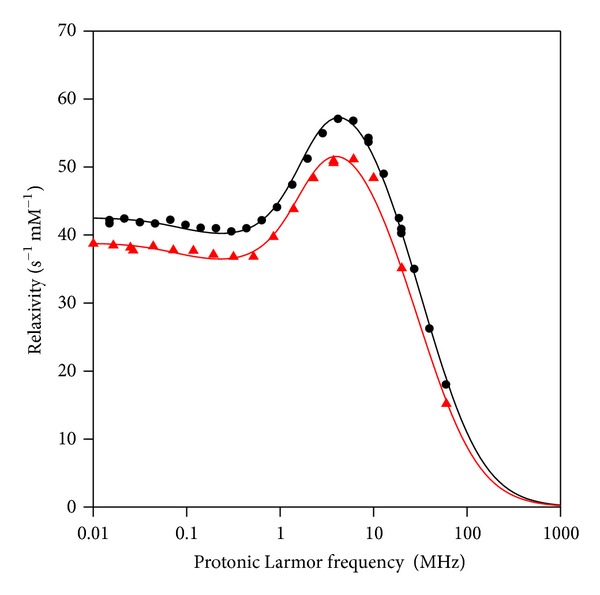
NMRD profiles of 2C peptide-USPIO-PEG (black circles) and USPIO-PEG (red triangles).

The 2C peptide-USPIO-PEG was prepared from iron oxide nanoparticles that were functionalized first with the peptide and then with an amino-PEG 750. These grafting steps do not change significantly the relaxometric properties of the nanoparticle. NMRD profiles showing the changes in relaxivity (1/T1) versus the applied magnetic field strength are given in Figure 5.

The physicochemical properties of the nanoparticles included a hydrodynamic size of 33 nm (determined by PCS), an *r*_1_  of 40.2 s^−1^ m M^−1^ and *r*_2_ of 81.6 s^−1^ m M^−1^ at 20 MHz, 37°C; an *r*_1_ of 17.9 s^−1^ m M^−1^ and *r*_2_  of 82.9 s^−1^ m M^−1^ at 60 MHz, 37°C.

The theoretical fitting of the NMRD profile according to the superparamagnetic relaxation model [20] gave a magnetization Msat of 55.82 Am^2^/kg and a radius of 5.76 nm. The vectorization with peptides and PEG does not change significantly the magnetic properties of the nanoparticles.

### 3.4. Immunohistochemical Assay

#### 3.4.1. TNF-*α* Immunodetection with a Polyclonal Antibody in a Mouse Model of Hepatitis

To confirm the specific affinity of the phage display-selected peptide PATLTSL, called peptide 2, to TNF-*α*, the proinflammatory cytokine was targeted in a well-known mouse model of hepatitis.

**Figure 6:**
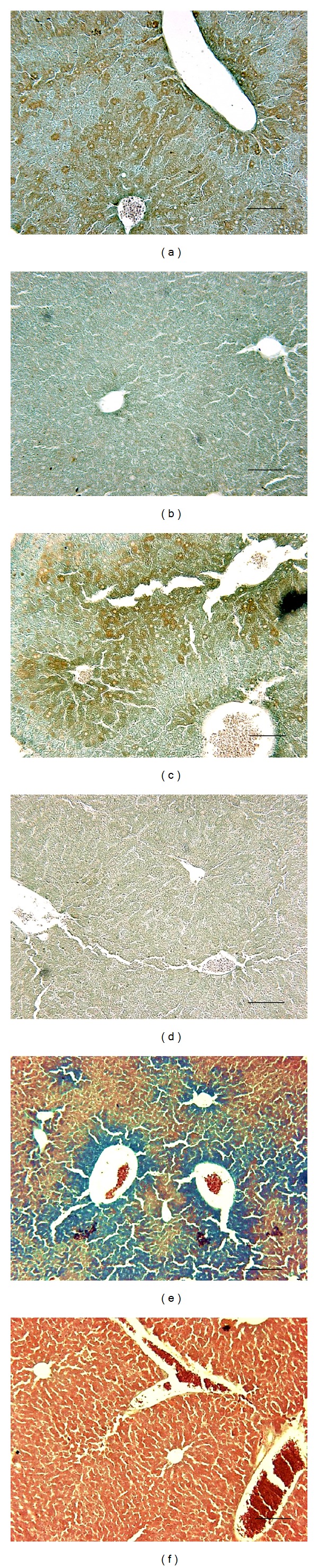
TNF-*α* detection in liver sections of ConA-treated mice.** **TNF-*α* was first detected with a polyclonal anti-TNF-*α* antibody in liver sections of ConA-treated mice by a brown coloration mainly around sinusoids and blood vessels (a) as compared to untreated-liver sections (b). TNF-*α* was then detected with the biotinylated 2C peptide (c) by a similar brown coloration located around sinusoids and blood vessels on liver sections of treated mice. The sections of healthy liver did not show such a staining (d). The 2C peptide-USPIO-PEG also allowed for the detection of the TNF-*α* cytokine on liver sections of treated mice (e), while it was not detectable on healthy liver sections (f). Scale bar: 100 *μ*m.

TNF-*α* was detected with a polyclonal anti-TNF-*α* antibody on liver tissue sections (Figures 6(a) and 6(b)) and was mainly detected around hepatic sinusoids 2 hours after i.v. ConA injection.

#### 3.4.2. TNF-*α* Detection by the Phage Display-Selected 2C and 2L Peptides

The affinity of the phage display-selected 2C and 2L peptides for TNF-*α* was evaluated by immunohistochemistry on liver sections obtained from mice treated with ConA. The cyclic form of peptide 2 produced a similar staining to that obtained with the polyclonal anti-TNF-*α* antibody (Figures 6(c) and 6(d)). On the contrary, the linear form did not yield such a result.

#### 3.4.3. TNF-*α* Detection with the Phage Display-Selected 2C Peptide Grafted to USPIO-PEG

After proving the affinity of the biotinylated 2C peptide on liver sections, this phage display-selected peptide was grafted to USPIO-PEG to study the affinity of the vectorized probe for the target. USPIO was detected with the Prussian blue iron staining method. A blue coloration indicated the presence of iron around sinusoids and blood vessels on ConA-treated liver sections (Figure 6(e)), similar to the results obtained with the biotinylated form of the 2C peptide. Healthy liver did not show any blue coloration (Figure 6(f)), suggesting that the vectorized nanoparticles were able to bind their target in the liver sections of ConA-treated mice. The selected peptide did not lose its affinity for TNF-*α* after grafting to USPIO.

## 4. Discussion

Inflammation is the first response of the immune system to infection, injury, or irritation. During the acute phase of the inflammatory process, some molecular and cellular changes occur like the accumulation of fluid, inflammatory cells, and soluble mediators. Many of these soluble mediators regulate the activation of resident cells such as monocytes, lymphocytes, or neutrophils, which results in the systemic response to the inflammatory process. These mediators include cytokines, inflammatory lipid metabolites, arachidonic acid derivatives (prostaglandin, leukotrienes), vasoactive amines like histamine or serotonin, and cascades of soluble proteases/substrates (clotting, complement, and kinin pathways), which generate numerous proinflammatory peptides. Among these factors, cytokines play key roles in mediating inflammatory reactions, especially IL and TNF, which are potent and primary inflammatory molecules mediating acute inflammation.

TNF-*α* is the most common proinflammatory cytokine secreted during the inflammatory process. It is involved in both acute and chronic inflammation and has a key position in the pathogenesis of various infectious and inflammatory diseases. It is abundantly present in inflammatory areas in two forms, a soluble and a transmembrane precursor forms. These characteristics make TNF-*α* a good candidate for inflammation targeting.

In the present study, we used the phage display technology to identify small peptide vectors for MRI detection of inflammation.

Results showed two phage displayed peptides having a better affinity for the target (TNF-*α*) among a selection of 28 phage clones. To optimize peptide target specificity, phage displayed peptides were incubated with TNF-*α* with an increasing concentration of destabilizing detergent during each round of selection. A competition test with an anti-TNF-*α* antibody was also performed to validate the specific interaction between peptides and their target.

Finally, the two best peptides were synthesized in a linear and a cyclic form, and *K*_d_* values were evaluated. The lowest *K*_d_* values of free peptides compared to phage displayed peptides might be explained by a multivalency effect allowed by the five peptide copies displayed by phages [22, 23]. However, it has been reported that a conformation constraint induced by disulfide cyclization of peptides could attenuate this affinity-decreasing effect [24]. The biotinylated linear and cyclic forms of peptide 2 were then chosen for histological studies on a well-known mouse model of hepatitis. TNF-*α* was mainly detected around hepatic sinusoids 2 hours after i.v. ConA injection, in accordance with previously reported data [12, 21].

The best result was obtained with the cyclic form of peptide 2 (called 2C peptide) which allowed detecting TNF-*α* as efficiently as the anti-TNF-*α* antibody on liver histological sections. The cyclization gives the peptide a spatial conformation that may facilitate its interaction with the target, thus allowing better TNF-*α* staining results than those obtained with the linear form of the same peptide.

Finally, the 2C peptide was grafted to iron oxide nanoparticles that were evaluated on liver sections. Results obtained with the new specific iron oxide nanoparticles were compared to anti-TNF-*α* antibody and to the synthesized biotinylated 2C peptide experiments performed on the ConA-treated mice liver sections. The specific 2C peptide-USPIO-PEG showed the same labeling as the antibody and the biotinylated 2C peptide on histological liver sections, showing that the USPIO-PEG, thanks to the TNF-*α* specific 2C peptide linked to their surface, allowed for the staining of this proinflammatory cytokine in a ConA-induced mouse model of hepatitis.

At present, some inflammatory diseases like arthritis, asthma, atherosclerosis, Crohn's disease, or neurodegenerative diseases are not well understood and consequently not well diagnosed. Molecular imaging, which uses small probes (e.g., MRI contrast agents) vectorized to an injured area, offers new tools for diagnosing idiopathic diseases [25–27]. Increasingly, studies are focusing on new targeted approaches to the detection and treatment of inflammation [28–30].

The aim of this work was thus to develop specific iron oxide probes by vectorization with phage display-selected heptapeptides to offer a new tool for the MRI diagnosis of pathologies with an abundant inflammatory process. In this molecular approach of MRI, magnetic nanoparticles are optimized to reach a specific target, known to be a characteristic and abundant molecular feature of a certain disease. In this work, TNF-*α* was chosen as a target to detect inflammation. The phage display-selected 2C peptide and the specific 2C peptide-USPIO-PEG seemed to be able to detect inflammation by interacting with TNF-*α* in ConA-treated mice liver sections.

According to these results, the 2C peptide is appealing for molecular imaging applications. It can, for instance, be used as a vector for magnetic probes like iron oxide nanoparticles or gadolinium complexes, providing a new targeted agent for the detection of inflammation in MRI.

## Figures and Tables

**Table 1:** Biochemical properties of the selected peptide clones (from http://www.expasy.ch/tools/protparam.html).

Clone	Sequence	T_1/2_ (hours)	pI
**C2-R3**	**C-GSKTQAP-C**	**30**	**8.06**
C18-R3	C-GLPWLST-C	30	5.51
**C22-R3**	**C-STPHNLG-C**	**1.9**	**6.72**
C25-R3	C-LATGNQI-C	5.5	5.51
C30-R3	C-PATLTSL-C	20	5.51
**C33-R3**	**C-HGAPNRL-C**	**3.5**	**8.08**
**C41-R3**	**C-RPPIGAF-C**	**1**	**8.07**
**C42-R3**	**C-TSQSQHM-C**	**7.2**	**6.72**
**C45-R3**	**C-GPAVYMK-C**	**30**	**8.05**
C46-R3	C-QGDLPGY-C	0.8	3.80
**C32-R4**	**C-SPHTTIA-C**	**1.9**	**6.72**
C33-R4	C-EPFAGRS-C	1	5.99
C35-R4	C-TSPLPGT-C	7.2	5.51
C46-R4	C-SYEAHQT-C	1.9	5.24
**C47-R4**	**C-NNPLKSL-C**	**1.4**	**8.06**

T_1/2_: half-life theoretically estimated in mammalian reticulocytes *in vitro* and based on the N-end rule [20].

pI: isoelectric point which is the pH at which a particular molecule or surface carries no net electrical charge.
